# Efficacy of the latest new stimulation patterns of spinal cord stimulation for intractable neuropathic pain compared to conventional stimulation: study protocol for a clinical trial

**DOI:** 10.1186/s13063-023-07637-x

**Published:** 2023-09-23

**Authors:** Takafumi Tanei, Satoshi Maesawa, Yusuke Nishimura, Yoshitaka Nagashima, Tomotaka Ishizaki, Masahiko Ando, Yachiyo Kuwatsuka, Atsushi Hashizume, Shimon Kurasawa, Ryuta Saito

**Affiliations:** 1 Department of Neurosurgery, Nagoya University Graduate School of Medicine, 65 Tsurumai-Cho, Showa-Ku, Nagoya, Aichi 466-8550 Japan; 2 Department of Advanced Medicine, Nagoya University Graduate School of Medicine, 65 Tsurumai-Cho, Showa-Ku, Nagoya, Aichi 466-8550 Japan; 3 Department of Clinical Research Education, Nagoya University Graduate School of Medicine, 65 Tsurumai-Cho, Showa-Ku, Nagoya, Aichi 466-8550 Japan

**Keywords:** Spinal cord stimulation, Neuropathic pain, Differential target multiplexed, Fast-acting subperception therapy

## Abstract

**Background:**

Spinal cord stimulation (SCS) is one of the neuromodulation therapies for chronic neuropathic pain. The conventional paresthesia-based SCS involves the application of tonic stimulation that induces a sense of paresthesia. Recently, new SCS stimulation patterns without paresthesia have been developed. Differential target multiplexed (DTM) stimulation and fast-acting subperception therapy (FAST) stimulation are the latest paresthesia-free SCS patterns.

**Methods:**

A single-center, open-label, crossover, randomized clinical trial to investigate the superiority of SCS using the latest new stimulation patterns over conventional tonic stimulation for neuropathic pain is planned. This study consists of two steps: SCS trial (first step) and SCS system implantation (second step). In the SCS trial, participants will be randomly assigned to 4 groups receiving stimulation, including tonic, DTM, and FAST. Each stimulation will then be performed for 2 days, and a visual analog scale (VAS) for pain will be evaluated before and after each stimulation pattern. A stimulation-off period for 1 day is set between each stimulation pattern to wash out the residual previous stimulation effects. Pain improvement is defined as more than 33% reduction in the pain VAS. The primary analysis will compare pain improvement between the new stimulation patterns and the conventional tonic stimulation pattern in the SCS trial. The secondary outcomes will be evaluated as follows: (1) the relationships between causative disease and improvement rate by each stimulation pattern; (2) comparison of pain improvement between the DTM and FAST stimulation patterns in all cases and by causative disease; (3) changes in assessment items preoperatively to 24 months after the implantation; (4) preoperative factors associated with long-term effects defined as continuing for more than 12 months; and (5) adverse events related to this study 3 months after the implantation.

**Discussion:**

This study aims to clarify the effectiveness of the latest new stimulation patterns compared to the conventional tonic stimulation. In addition, which stimulation pattern is most effective for which kind of causative disease will be clarified.

**Trial registration:**

Japan Registry of Clinical Trials (jRCT) 1,042,220,094. Registered on 21 November 2022, and last modified on 6 January 2023. jRCT is an approved member of the Primary Registry Network of WHO ICTRP.

## Background

Neuropathic pain is caused by lesions in somatosensory pathways of the peripheral or central nervous system. The estimated prevalence of neuropathic pain ranges from 1 to 7% [1]. Pharmacological treatment of neuropathic pain consists mainly of the use of pregabalin, antidepressants, and opioids [2]. However, many patients do not respond to pharmacological treatment. For this reason, non-pharmacological approaches such as neuromodulation therapies have been developed [3]. Spinal cord stimulation (SCS) is one of the neuromodulation therapies that has been used for decades to treat chronic neuropathic pain [4–7]. The conventional SCS consists of the application of tonic stimulation that induces a sense of paresthesia. It is essential for the amelioration of pain that the elicited paresthesia overlaps the painful area [8]. The pain relief mechanism of SCS using conventional tonic stimulation is based on the gate control theory [9, 10]. According to the theory, SCS induces activation of spinal GABAergic interneurons in the dorsal horn and descending pain-inhibitory pathways [9, 10].

Since the 2010s, several new SCS stimulation patterns without paresthesia have been developed [11–14]. These new stimulation patterns are described as “paresthesia-free” or “subperception” SCS. Differential target multiplexed (DTM) stimulation (Medtronic Inc., Minneapolis, MN, USA) and fast-acting subperception therapy (FAST) (Boston Scientific, Marlborough, MA, USA) stimulation are among the latest paresthesia-free SCS patterns, which have been available in Japan since 2021. It has been suggested that SCS with the latest stimulation patterns has higher pain relief effects than the conventional tonic stimulation [15–17]. Furthermore, the latest new stimulation patterns may be effective in cases where the conventional tonic stimulation cannot be continued because of the discomfort of the paresthesia [18–20]. Meanwhile, which stimulation is most effective for which kind of neuropathic pain remains unclear.

Whereas all SCS devices can provide conventional tonic stimulation, the new stimulation patterns are device-dependent, with each manufacturer providing its own stimulation pattern. This means that only one of the new stimulation patterns can be applied to a patient after SCS system implantation. For this study, a method that makes it possible to apply both DTM and FAST stimulation patterns to one patient during the SCS trial is performed: the Medtronic percutaneous leads are inserted and external state which applies DTM stimulation, and a Boston Scientific cable is connected to the external leads to apply FAST stimulation.

The first clinical question is whether SCS using the latest stimulation patterns for neuropathic pain provides more pain relief than the conventional tonic stimulation. The second clinical question is which kind of stimulation provides the greatest pain relief effect for which kind of neuropathic pain. The hypothesis to be tested in this study is that SCS with the latest stimulation patterns provides significantly more pain relief than the conventional tonic stimulation for patients with neuropathic pain.

## Methods/design

### Study design

This is a single-center, open-label, crossover, randomized, superiority clinical trial.

### Patient population

Patients will be selected based on inclusion and exclusion criteria as shown below. The main criterion for enrollment is intractable neuropathic pain without a history of spinal surgery at the site where the SCS lead will pass or be placed. Patients will be recruited from new patients treated according to standard clinical practice at Nagoya University Hospital.

### Inclusion criteria

- Intractable neuropathic pain resistant to drug treatment using more than one drug.
- Age ≥ 18 years.
- Pain visual analog scale (VAS) score > 40.
- Written, informed consent.

### Exclusion criteria

- Anti-cancer therapy.
- History of drug abuse.
- Histories of spine or spinal cord surgery at the site through which the spinal cord stimulation lead passes or will be placed.
- Local and general anesthesia cannot be performed.
- Conditions resulting in high surgical risk, such as unstable angina pectoris and end-stage liver disease presenting with hepatic encephalopathy.
- Poorly controlled diabetes mellitus (HbA1c ≥ 9%).
- Serious concomitant diseases (liver disease, kidney disease, heart disease, lung disease, blood disease, brain disease, etc.)
- Pregnant or potentially pregnant.
- Considered inappropriate by the head of research or researcher allocating patients.

### Who will take informed consent?

Potential participants will be identified from among patients visiting Nagoya University Hospital. After clinical research physicians assess potential participants for the inclusion and exclusion criteria, they will be given study information in detail of the purpose, procedure, potential risks, and benefits of the trial. Eligible patients will need to sign two informed consents, one held by the researcher and the other by the patient, if they wish to participate. The right of a participant to refuse to participate in this trial without giving reasons for the decision will be respected.

### Additional consent provisions for collection and use of participant data and biological specimens

The consent form received from the patients includes their agreement to use the data in related future studies. Of course, patients can only agree to the current study and not accept future studies.

### Study procedures

This trial consists of two steps. The first step is an SCS trial, and the second step is SCS system implantation. In the SCS trial, two cylinder-type leads (Model 977A190; Medtronic Inc.) will be inserted under local anesthesia and connected to an external stimulation device, and intraoperative stimulation will be performed to confirm the area of paresthesia induction. The leads will be placed at vertebral levels of the paresthesia covering the painful area and then directly sutured to the skin at the puncture sites without a skin incision. During the SCS trial, three stimulation patterns, tonic, DTM, and FAST, will be applied. After the SCS trial ends, the inserted leads will be removed in all cases. If pain relief effects are obtained in the SCS trial, the cases will proceed to the second step. More than 1 month after the SCS trial, new cylinder-type leads and an implantable pulse generator (IPG) will be implanted under general anesthesia referring to the previous X-ray of the SCS trial lead placement. The type of implanted devices will be determined as follows: leads (Model 977A190; Medtronic Inc.) and IPG (Intellis; Medtronic Inc.) will be implanted if the DTM stimulation pattern is effective; leads (Linear ST lead; Boston Scientific) and IPG (WaveWriter Alpha 32; Boston Scientific) will be implanted if FAST or tonic stimulation patterns are effective.

### Method of SCS trial

**Fig. 1:**
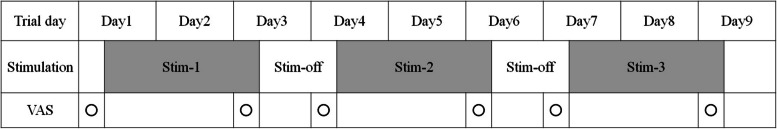
The schedule of stimulation patterns and timing of pain assessments. The SCS trial period will last 9 days and consist of 3 stimulation-on periods and 2 stimulation-off periods. The initial stimulation will be started 1 day after SCS lead insertion. The pain relief effect will be assessed using a VAS scale 6 times: pre/post Stim-1, pre/post Stim-2, and pre/post Stim-3. After the assessment of post Stim-3, the SCS leads will be removed

The SCS trial period will last 9 days and consist of 3 stimulation-on periods and 2 stimulation-off periods (Fig. 1). The initial stimulation will be started one day after SCS lead insertion. The pain relief effect will be assessed using a VAS scale 6 times: pre/post Stim-1, pre/post Stim-2, and pre/post Stim-3. After the assessment of post Stim-3, the SCS leads will be removed. The order of stimulations will be set differently among 4 groups. The order of stimulations is pre-determined for each group and the order of stimulations of each group is shown in Table 1. For example, the order of group 1 will be Tonic stimulation (Stim-1), DTM stimulation (Stim-2), and FAST stimulation (Stim-3). Each stimulation pattern will be performed for 2 days. There will be a 1-day stimulation-off period between each stimulation pattern (Stim-off). The participants will be randomly assigned to one of the 4 groups at the time of study registration. In other words, the order of stimulations is determined when the participants are assigned to the group.

**Table 1:** The order of stimulations for each group

	Stim-1	Stim-2	Stim-3
Group1	Tonic	DTM	FAST
Group2	DTM	Tonic	FAST
Group3	Tonic	FAST	DTM
Group4	FAST	Tonic	DTM

*DTM* differential target multiplexed, *FAST* fast-acting subperception therapy

### Setting of each stimulation pattern

Tonic stimulation is a conventional stimulation pattern that delivers mild electrical pulses and elicits paresthesia. Stimulation parameters include frequency, pulse width, and voltage. Frequency (10–100 Hz) and pulse width (40–300 μs) will be set by clinical research physicians so that the patient feels the paresthesia as a comfortable sensation. After setting both frequency and pulse width, the voltage will be adjusted by the patient using a remote control.

DTM stimulation is one of the latest paresthesia-free stimulation patterns that delivers multiple electrical signals and stimulates multiple locations without paresthesia. DTM stimulation consists of one stimulation signal on the upper side of the leads, called the base program, and multiple stimulation signals on the lower side of the leads, called the prime program. The base program is set at a frequency of 50 Hz, pulse width of 200 μs, and voltage of approximately 70% of the paresthesia threshold. Three prime programs are set at the lower side of the base program. The prime program is set at a frequency of 300 Hz, pulse width of 170 μs, and voltage of approximately 65% of the paresthesia threshold. DTM stimulation delivers electrical signals from the base and three prime programs. Patients do not use a remote control during DTM stimulation.

FAST stimulation is a latest paresthesia-free stimulation pattern that delivers two symmetrical biphasic waveforms to the leads with a frequency of 90 Hz and a pulse width of 210 μs. Each wave is a rectangular phase of the charge-balanced stimulation cycle. During the first rectangular phase, a negative current is injected through negatively configured leads, and a positive current is injected through positively configured leads. During the second rectangular phase, the polarities are reversed to achieve charge balance. Then, positive and negative reversal stimulations are repeated. The stimulation power is lowered to approximately 30% of the paresthesia threshold. Patients do not use a remote control during FAST stimulation.

### Criteria for discontinuing or modifying allocated interventions

Any patients requesting to end their participation in the study can be withdrawn from the study regardless of the stage they have reached in the study process. Patients found to be pregnant or those judged ineligible to continue participating in the study by the investigators will also be withdrawn from the study.

### Strategies to improve adherence to interventions

All treatments will be administered to participants during their stay in the hospital by attending surgeons. Therefore, participants’ adherence to interventions is assured.

### Relevant concomitant care permitted or prohibited during the trial

All other treatments will be allowed.

### Provisions for post-trial care

Any patients who suffer harm from trial participation will be covered by the Japanese public healthcare system.

### Clinical assessments

On enrollment in this trial, clinical research physicians will obtain information from the patients including age, sex, past history, current medication, causative disease of pain (central post-stroke pain, post–spinal cord injury pain, failed back surgery syndrome, complex regional pain syndrome, post-herpetic neuralgia, diabetic neuropathic pain, peripheral arterial disease, or others), lesion site causing pain (central or peripheral), location of pain (arm, leg, lower back, back, chest, face), laterality of pain (left, right, midline), degree of paralysis (none, mild, moderate, severe), sensory disturbance (hypoesthesia, allodynia, numbness), and duration of disease. During the SCS trial, the degree of pain will be evaluated using a VAS according to the plan (Fig. 1). After SCS system implantation, assessment items will be evaluated according to the plan, including the degree of improvement with respect to pain relief and mental state (Table 2).

**Table 2:**
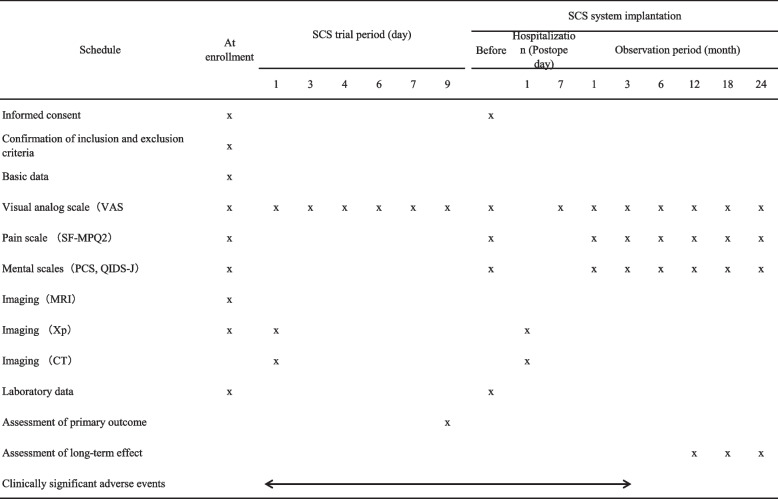
Summary of observations, assessment items, and schedule of assessments

*VAS* visual analog scale, *SF-MPQ-2* Short-Form McGill Pain Questionnaire-2, *PCS* Pain Catastrophizing Scale, *QIDS-J* Quick Inventory of Depressive Symptomatology, *MRI* magnetic resonance image, *Xp* X-ray picture, *CT* computed tomography, *SCS* spinal cord stimulation

### Assessment items

Assessments of pain relief will be performed using the VAS and Short-Form McGill Pain Questionnaire-2 (SF-MPQ-2), and mental state will be assessed using the Pain Catastrophizing Scale (PCS) and Quick Inventory of Depressive Symptomatology (QIDS-J).

### Imaging evaluation

Before the SCS trial, whole-spine magnetic resonance imaging and X-ray examinations will be performed to rule out any abnormal findings. After the SCS trial and SCS system implantation, spinal X-ray and computed tomography examinations will be performed to confirm the location of the SCS leads and to rule out postoperative complications.

### Primary outcome

The primary analysis will compare the pain improvement between the new stimulation patterns (DTM or FAST) and the conventional tonic stimulation in the SCS trial. The pain improvement of each stimulation pattern in the SCS trial is defined as effective when the VAS value decreases by 33% or more compared to the VAS value before the start of each stimulation pattern. The primary outcome will be analyzed at the end of the SCS trial.

### Secondary outcomes

The following secondary outcomes will be analyzed at the end of the SCS trial: (1) the relationship between causative disease and improvement rates by each stimulation pattern and (2) comparison of the improvement rates between DTM and FAST stimulation patterns in all cases and by causative disease.

After SCS system implantation, the efficacy of SCS is defined as when the VAS value decreases by 33% or more compared to the VAS value before the implantation. Long-term effect is defined as continuing the efficacy of SCS for more than 12 months. The following secondary outcomes will be assessed for six times during 24 months’ follow-up periods after the implantation (Table 2): (3) changes in assessment items including the VAS, SF-MPQ-2, PCS, and QIDS-J preoperatively, and at 1, 3, 6, 12, 18, and 24 months after the implantation; (4) preoperative factors associated with the long-term effects; and (5) adverse events related to this study 3 months after the SCS system implantation. Data will be presented as mean and standard deviation for continuous variables (including VAS score, SF-MPQ-2 score, PCS score, and QIDS-J score).

#### VAS

The VAS is a basic self-reported measure for assessing pain intensity. It is a horizontal line of 100 mm, with 0 mm (the left endpoint) denoting no pain and 100 mm (the right endpoint) indicating excruciating pain [21]. The participants will be requested to choose the location on the line that best indicates their pain level.

#### SF-MPQ-2

The SF-MPQ-2 consisted of 22 items to provide increased responsiveness [22]. Each item is evaluated with an 11-point scale (0 representing no pain and 10 representing the worst pain imaginable). The SF-MPQ-2 is divided into 4 subscales, including 3 sensory descriptors and 1 affective descriptor, as follows: continuous pain descriptors (6 items), intermittent pain descriptors (6 items), predominantly neuropathic pain descriptors (6 items), and affective descriptors (4 items). The total pain dimension parameter is the sum of the sensory and the affective dimension of the pain (score range: 0 to 220). A high score indicates a high level of pain.

#### PCS

The PCS is used to measure pain-related catastrophic thoughts and consisted of 13 items [23]. Each item is scored on a 5-point Likert scale; evaluations (0 = not at all to 4 = always) are made. The scale assesses catastrophic thinking/feeling regarding 3 components: rumination, magnification, and helplessness. The total score range is from 0 to 52 points, and a higher score indicates a higher level of pain-related catastrophic thoughts.

#### QIDS-J

The QIDS-J consists of 16 questions and plays a role in the diagnostic criteria for major depressive disorder [24]. These questions are divided into nine categories. Four concerned sleeping disorders, four appetite and body weight, two psychological disorders, and the remaining nine other topics. The total score range is from 0 to 48 points, and a higher score indicates a higher level of depressive disorder.

### Sample size

The study’s sample size is based on the expected difference of the percentage of patients achieving pain improvement between the new and conventional tonic stimulation patterns.

We assumed that the percentages of pain improvement will be 80% for the new stimulation patterns (DTM or FAST) [15, 17] and 50% for the conventional tonic stimulation pattern [25–28] by reviewing the literature. The required sample size was calculated to be 78 cases with a significance level of 0.05, a power of 80%, and 1:1 allocation. Each group (A, B, C, and D) will enroll 20 cases (40 conventional tonic stimulation and 40 new stimulation patterns in Stim-1). Thus, it is possible to secure a power of 0.8 even with the analysis of Stim-1 alone. In addition, considering the insufficient sample size caused by patient detachment during the research process, it is necessary to expand the target number of cases is 23 in each group, resulting in a total sample size of 92 patients in all groups. Assuming, based on past performance, that there are 2–4 cases enrolled in the SCS trial per month, 24–48 patients are expected to be enrolled annually. Therefore, the target number is expected to be reached in 2 to 4 years.

### Data management

Randomization will be performed centrally through the web-based system with a minimization procedure. The allocation sequence using the web-based system will be generated at the data center (Department of Advanced Medicine, Nagoya University Hospital). Enrolment of participants and assignment to interventions will be performed by TT. Registration, randomization, and data collection will be performed using an electronic data capture (EDC) system. Statistical analyses will be performed at the data center. Data collection is performed partially on paper source documents (patient questionnaires) and partially on electronic source documents (patient medical records containing surgical and hospitalization reports, registration of used devices). Data are entered into the electronic data capture system (REDCap) independently by one researcher and checked by another researcher. The RedCap is primarily a data collection tool that facilitates post-study analysis based on qualitative data. Access to the RedCap is strictly regulated and only with personal credentials. All data are monitored and verified via a tracking system.

### Concealment mechanism

The results of the allocation will be shown via the interactive web response system.

### Assignment of interventions: blinding

#### Who will be blinded

The randomization is single-blinded, i.e., participants do not know which group they are allocated to. Blinding of the researchers and data analysts is not possible because each stimulation pattern is not able to be completely concealed.

#### Procedure for unblinding if needed

Unblinding will not be needed because this study is single-blind.

#### Plans to promote participant retention and complete follow-up

Medical interviews and adjustments of the SCS parameters will be booked for all patients.

#### Confidentiality

The form used to code patients will be stored in a locked cabinet with logged access only available to the researchers and administrators responsible for the study.

#### Plans for collection, laboratory evaluation, and storage of biological specimens for genetic or molecular analysis in this trial/future use

Not applicable. No biological specimens will be involved in this study.

### Statistical analysis

The primary analysis will be performed after the completion of the SCS trial of the final registered participant. An analysis of the 12-month follow-up period data will be performed at the completion of the 12-month follow-up after the implantation of the final registered participant. Analyses of the 18- and 24-month follow-up period data will be performed at the completion of 24-month follow-up from the implantation of the final registered participant.

The primary outcome analysis will be as follows. Pain improvement will be compared between each stimulation using logistic regression analysis with explanatory variables of stimulation timing (Stim-1, Stim-2), treatment group (new stimulation patterns, conventional tonic stimulation), pre-VAS score for each stimulation pattern, and interaction between stimulation timing and treatment group. The carryover effect will be examined by testing the interaction terms with a significance level of 10%. If there is no carry-over effect, treatment groups will be compared with a significance level of 5%. If a carryover effect is observed, analysis will be performed with data from Stim-1 only.

The secondary outcome will be analyzed as follows:

1. Similar analyses of the primary outcome analysis will be performed for each causative disease.
2. Comparison of DTM and FAST stimulation patterns for pain improvement in Stim-3. Pain improvements will be compared between DTM and FAST stimulations using logistic regression analysis adjusted for the treatment group (DTM stimulation, FAST stimulation) and pre-VAS value. Similar analyses will be performed by causative disease.
3. Adjusted mean values and 95% confidence intervals of changes in assessment items including the VAS, SF-MPQ-2, PCS, and QIDS-J before the SCS system implantation, and at 1, 3, 6, 12, 18, and 24 months after SCS system implantation at each time point will be calculated using a linear mixed model with the interaction between treatment group and time point as a fixed effect. Analysis will be performed using a linear mixed model with the change rate and change amount of each indicator as outcome variables. The rate and amount of changes in each index at each evaluation time point will be compared between groups using fixed effects such as pretreatment value of each index, treatment group, evaluation time point, and interaction between the treatment group and evaluation time point. The 12-month analysis will be performed at preoperative, 1, 3, 6, and 12 months, and the 24-month analysis will be performed at 18 months and the final follow-up (24 months).
4. In participants with no missing VAS values preoperatively to 12 months after implantation, analyses of the prognostic factors related to the long-term effect of continuing 33% reduction in the pain VAS over 12 months will be performed using logistic analysis with the following explanatory variables: treatment group, pre-implantation VAS value, sex, age, causative disease, site of pain, degree of paralysis, degree of sensory disturbance, and disease duration.

A comparison between the new and conventional stimulation patterns will be performed with Stim-1and Stim-2. A comparison between DTM and FAST, which is the new stimulation pattern, will be performed with Stim-3. Therefore, this study is designed to compare the VAS improvement rates in different stimulation patterns, so it is not necessary to consider multiplicity.

#### Interim analyses

Interim analyses are not planned.

#### Methods in analysis to handle protocol non-adherence and any statistical methods to handle missing data

The primary outcome will be analyzed according to a modified-intention-to-treat principle: all randomized patients who underwent SCS lead insertion will be analyzed according to their group allocation, irrespective of the treatment they received. Patients with missing outcomes will be treated as not having met the criteria for pain improvement. Missing data will not be imputed. When the case is dropped or patient’s withdrawal, the reasons will be mentioned in detail.

#### Plans to give access to the full protocol, participant-level data, and statistical code

The study is registered at Japan Registry of Clinical Trials (jRCT 1042220094). At the end of the study, the full protocol, data, and analysis results will be available from the corresponding author upon reasonable request.

### Oversight and monitoring

#### Composition of the coordinating center and trial screening committee

Nagoya University will serve as the coordinating center. Only the investigators and members of the data center will have access to the anonymized data in the REDCap.

#### Composition of the data monitoring committee, its role, and reporting structure

Two participating researchers at Nagoya University Hospital will monitor the data. They have the responsibility of verifying patients’ eligibility, written, informed consent, compliance with the protocol, and accuracy of the data in REDCap.

#### Adverse event reporting and harms

Adverse events that may be expected in the study population include infection, hemorrhage, nerve damage, and device failure or misalignment. During hospitalization, adverse events will be systematically collected from clinical and radiological examination records and daily nursing reports in the electronic patient records. At the outpatient follow-up visits, adverse events will be collected based on the history and clinical examination. During the study, all adverse events will be recorded in REDCap. A serious adverse event is defined as death, or persistent or significant disability or incapacity that requires hospitalization or prolongation of existing hospitalization. Such serious and all other adverse events will be reported in the final manuscript of the study. Researchers will immediately report serious adverse events associated with the trial to the chief investigator. The chief investigator will then report serious adverse events to the director of the hospital and the principal investigator. Data about all serious adverse events will also be collected in REDCap.

#### Frequency and plans for auditing trial conduct

No audits are to be conducted in this study. SM and YN are the monitoring staff, and they are members of the trial team. The monitoring will be conducted by visits, e-mail, etc., at an appropriate frequency, checking the following items: (1) consent acquisition; (2) eligibility assessment; (3) observance of the study protocol; (4) presence or absence of diseases; (5) consistency between source documents and case reports; (6) confirmation of serious illnesses; (7) clinical study procedures; and (8) storage status of documents.

#### Plans for communicating important protocol amendments to relevant parties

Any protocol modifications will be reviewed by the Certified Review Board of Nagoya University Hospital and then registered at jRCT. All relevant information will be shared among the researchers.

#### Dissemination plans

The results of this study will be published in a peer-reviewed journal and presented at national and international medical congresses.

## Discussion

This is a single-center, open-label, crossover, randomized clinical trial to elucidate whether SCS using the latest new stimulation patterns for neuropathic pain provides more pain relief than the conventional tonic stimulation pattern, and which kind of stimulation provides the most pain relief effects for which kind of neuropathic pain.

When assessing the effects of multiple stimulation patterns, there are two main biases. One is the order of stimulation, and the other is residual previous stimulation effects. To eliminate the initial bias, participants will be randomly assigned to 4 groups with different orders of the stimulation patterns (tonic, DTM, and FAST). The order of stimulation will be assessed among 4 respective groups because the study design is a comparison of the latest stimulation patterns and the conventional tonic stimulation. To eliminate the second bias, a 1-day stimulation-off period is set before administering each stimulation pattern to wash out the previous stimulation effects. Since the electrodes are external during the trial, the trial period is set to be within 10 days to avoid the risk of infection. Therefore, the duration of each stimulation pattern is set to 2 days, and stimulation-off is set to 1 day.

In this study, patients with histories of spine or spinal cord surgery at the site through which the SCS lead passes or will be placed will be excluded. The reason is that such patients may have adhesions around past spinal surgeries that make it difficult to pass the cylinder-type leads. Such patients will be enrolled in our other concurrent prospective clinical study [29]. In the concurrent study, treatment success is defined as a greater than 33% improvement in pain relief. Therefore, this study also defines treatment success as a greater than 33% improvement in pain relief in order to maintain consistency between the two parallel studies.

When performing SCS to relieve neuropathic pain, it is common to first evaluate the pain-relieving effects using an SCS trial and then implant the SCS system [4–8]. This two-step procedure is not unique to this study and is actually the method adopted by many clinical practices. The SCS trial is an essential step particularly for central neuropathic pain, such as central post-stroke pain, because the efficacy of SCS for such pain is not yet considered sufficient [25–28]. When performing a fair assessment of SCS efficacy, it is necessary to evaluate the effect in all cases, including non-responders. The primary and secondary outcomes (1) and (2) of this study will be analyzed at the end of the SCS trial to include all cases. However, the secondary outcomes (3) and (4) will be analyzed in relation to the changes in each assessment item over time only for responders, not including non-responders. Therefore, these secondary outcomes are not fair analyses and should be used for reference. To accurately verify long-term SCS effects and identify long-term efficacy predictors, a well-designed, prospective, randomized study with limited causative diseases and stimulation patterns will be necessary.

## Trial status

This manuscript is based on the protocol (version 2, last updated on October 5, 2022). The first patient was recruited on February 20, 2023. Recruitment will be completed by March 2028.

## Data Availability

Any data required to support the protocol can be supplied on request.

## Abbreviations

DTM: Differential target multiplexed
EDC: Electronic data capture
FAST: Fast-acting subperception therapy
IPG: Implantable pulse generator
jRCT: Japan Registry of Clinical Trials
PCS: Pain Catastrophizing Scale
QIDS-J: Quick Inventory of Depressive Symptomatology
REDCap: Research Electronic Data Capture
SCS: Spinal cord stimulation
SF-MPQ-2: Short-Form McGill Pain Questionnaire-2
VAS: Visual analog scale
